# Hemorrhagic Fever

**Published:** 1893-09

**Authors:** O. B. Bush

**Affiliations:** Arlington, Ga.


### HEMORRHAGIC FEVER.

By O. B. BUSH, M. D.,
Arlington, Ga.

Hemorrhagic fever is one of the most fatal fevers that we have to contend with, being next in fatality to yellow fever.

There are but few physicians that know how to treat this dreadful malady. This being the case and thinking that a short article on the subject will be of interest, I will endeavor to discuss briefly its etiology, diagnosis, prognosis and treatment.

*Etiology.*—Hemorrhagic fever is not infrequently a sequel of a severe attack of bilious fever, and it is frequently caused from *mercury,* the patient having an idiosyncrasy for mercury (calomel), which is necessary in *every* case of bilious fever; but generally speaking, hemorrhagic fever is caused from a specific germ, the name of which is known as malaria or miasma, which is inhaled from the low, marshy places with which this country (South-west Georgia) abounds. It (malaria) is also taken into the system through drinking water which some people (generally of the lower class) use, being compelled, on account of wells drying up, to get water from a creek or pond. This germ is carried into the stomach, and from there to the kidneys and all over and through the general system, thereby causing hemorrhage of the kidneys.

*Diagnosis and Symptoms.*—There is a loss of appetite, a furred tongue, the fur being of a whitish yellow, the bowels constipated, some pain may occur in the region of the stomach; there is considerable nausea and some vomiting, the color of the vomit being of a yellow color containing bile pigment. The liver is somewhat enlarged and tender.

The skin takes on an icteroid appearance, the sclerotic becomes yellow, the temperature ranges from 103° to 105°, the head sometimes aches and vertigo may probably occur; the spirit is depressed (from the knowledge of the patient of the fatality of this fever). The urine is almost entirely blood, the pulse runs from 120 to 140 to the minute. If the hemorrhage from the kidneys is not arrested in two or three days there will be complete exhaustion and emaciation.

*Prognosis.*—The mortality rate does not differ radically in the different epidemics that occur as does the mortality of yellow fever. The death rate in this fever is about two out of every three, or about sixty-six per cent.; this occurs not because the disease is so fatal within itself, but because it is made so on account of the ignorance of the attending physician, as will be seen in the treatment. The average duration is from five days to two weeks. If you can hold your patient up from six to thirty-six hours, as the case may be, after the first hemorrhage, the prognosis is generally favorable; the most favorable signs are a decrease in temperature, a quiet stomach and a diminution in the amount and frequency of the hemorrhages. But when the temperature is high and uncontrollable, the stomach nauseated and black vomit sets up, and the amount and frequency of the hemorrhages (which is very often from one quart to one-half gallon every fifteen, twenty or thirty minutes) increase the prognosis is very unfavorable. You had better withhold a prognosis until you can give a positive one.

*Treatment.*—There is no need of prophylaxis being used here, as this fever is caused solely from malaria, thought it is recommended by some of the authors. The treatment depends a great deal upon the condition and stage you find your patient in upon arrival. If the hemorrhage has not already set up, and the fever is very high (which is very sure to be the case, as you are not likely to be called until the cold stage has passed), the remedies needed are nitre and paregoric and viratrum (fever drops); this being given to run the fever down and to stay the heart's action to some extent. The natural inclination would be to give mercury, but not so; give nothing to devitalize the patient, as the hemorrhage might set up and, of course, it would be worse than if you had not given the mercury, because both (the calomel and hemorrhage) are very devitalizing. But if the hemorrhage has already set up when you get there, the first thing to be done is to check the hemorrhage if possible. To do this the best remedies to use are laudanum, buchu, nitre and tannic acid combined and given every two hours until hemorrhage ceases. In the meantime use, or if in use continue its use, the febrifuge to control the fever. Give stimulants to keep your patient stimulated, and nourishments to sustain your patient. If vomiting or nausea occurs give one-eighth of a drop of carbolic acid every half or every hour (as indicated) until nausea ceases. Now, the question naturally arises, what are you going to do to get rid of the cause of this trouble (malaria)? Give a solution of nitro-muriatic acid every four hours; that will answer all purposes until the patient can sufficiently recover to allow a tonic, then put him on a tonic of iron (mur. tr.), quinine and rhubarb. Authors of ancient times recommended, and do yet, bloodletting, but it is not only wrong in theory but decidedly wrong in practice. If constipation occurs give a mild cathartic, oil or salts; or if, on the other hand, they become troublesome, diarrhea sets up, give pill of opium and bismuth. Give no calomel, as it will certainly do no good, besides will do the greatest of harm—kill the patient.

